# Efficacy of Estradiol Cream for Venipuncture Pain in Obese Female Patients: A Pilot Study

**DOI:** 10.7759/cureus.23557

**Published:** 2022-03-28

**Authors:** Abhinay Jayanthi, Ghanshyam Yadav, Reena ., Amrita Rath

**Affiliations:** 1 Anesthesiology, Institute of Medical Sciences, Banaras Hindu University (BHU), Varanasi, IND

**Keywords:** vas, analgesia, estradiol, emla, venipuncture

## Abstract

Introduction: Venipuncture is often a painful procedure causing significant anxiety, distress, and psychological consequences. We evaluated the efficacy of estradiol cream and compared it with a eutectic mixture of local anesthetics (EMLA) cream for alleviation of venipuncture pain and to make cannulation easy in female obese patients.

Materials and methods: The clearance from the Institutional Ethical Committee as well as prior written and informed consent were obtained from the participants. A total of 105 obese female adult patients aged between 25 and 64 years belonging to the American Society of Anesthesiologists (ASA) physical status I and II with body mass index (BMI) > 30 kg/m^2^ were included in our study. The study participants were randomly allocated into three groups: In group I, a placebo cream was applied; in group II, estradiol cream was applied, and in group III, EMLA cream was applied. Any abnormal sensation at the site of application of the cream was noted and followed up at 0, 2, and 6 hours for the same. The primary outcome was the measurement of the severity of the pain experienced during venipuncture using the visual analog scale (VAS). Ease of cannulation was our secondary outcome. VAS was compared with the Z test. Statistical Package for the Social Sciences (SPSS) v16.0 software (SPSS Inc., Chicago) was used for statistical analysis. A p-value of <0.05 was considered statistically significant.

Result: The final analysis was carried out on 25 patients in group I, 27 patients in group II, and 33 patients in group III. There was no significant improvement in the ease of cannulation in group II when compared to group I. The mean VAS was similar in group I and group II, whereas it was significantly reduced in group III (p < 0.05).

Conclusion: EMLA cream was found to significantly reduce the pain of venipuncture in comparison to placebo and estradiol cream. There is no beneficial effect of estradiol cream in reducing the pain from venipuncture or in ease of cannulation compared to placebo.

## Introduction

Venipuncture, although a minor procedure that is done in routine anesthesia practice, is often a painful procedure with the potential to cause significant anxiety, distress, and psychological consequences [1,2]. It is a growing practice among healthcare providers to use topical analgesics before venous cannulation to achieve a pain-free and comfortable hospital stay for patients [3].

Among the 33 low morbidity clinical outcomes, the discomfort caused by venous catheter insertion was ranked fifth by a study conducted to know the perspective of a panel of expert anesthesiologists to ascertain which clinical anesthesia outcomes are common and important to avoid during the practice of anesthesia [4]. Various pharmacological and non-pharmacological techniques of minimizing the pain of venipuncture have been already described in previous literature such as application of a eutectic mixture of local anesthetics (EMLA) cream, diclofenac patch, ibuprofen, piroxicam, lignocaine, ethyl chloride spray, ice, distraction tactics, cough trick, Valsalva, inflation of the balloon, nitrous oxide, and oral sucrose with variable results [5-15]. Estradiol has antinociceptive and vasodilating properties [16,17]. Obesity is known to be associated with difficult venous cannulation [17,18]. To add to it, females are more pain-sensitive than males [19]. In the present study, we evaluated the efficacy of estradiol cream and compared it to EMLA cream in attenuating venipuncture pain and making cannulation easy in female obese patients.

## Materials and methods

After getting approval from the Institute’s Ethical Committee and obtaining written informed consent from patients, this prospective, randomized, double-blind, and placebo-controlled study was conducted from the period 2014 to 2020 at the Institute of Medical Sciences, Banaras Hindu University (CTRI/2014/11/005162). A total of 105 female adult patients aged between 25 and 65 years belonging to the American Society of Anesthesiologists (ASA) physical status I and II with body mass index (BMI) > 30 kg/m^2^, who were hemodynamically stable and undergoing elective surgeries under general anesthesia, were included in our study. Patients with a history of hypertension, diabetes, liver disease, acute or chronic renal disease, peripheral neuropathy, known psychiatric disorders, abnormal skin conditions at the desired site of venous puncture, patients who were undergoing neurosurgical and cardiovascular surgical cases, and pregnant patients were excluded from the study. Also, patients on antihypertensive drugs, sedatives, hypnotics, antidepressants, long-term analgesics, drugs with effects on the nervous system and patients who were already taking estradiol cream or having an allergy to estradiol were excluded from the study.

Assuming that this intervention will reduce the venipuncture pain by 20% as assessed by visual analog scale (VAS), a power analysis, with α = 0.05; β = 0.20, shows that we needed to enroll 32 patients in each group. To compensate for dropouts at any stage, we enrolled 35 patients in each group.

The patients were randomly assigned into three equal groups with the help of computer-generated random numbers. In group I (control): a placebo cream was applied. In group II (estradiol): the estradiol cream (containing 5% w/w, manufactured by Geno Pharmaceutical Ltd., Goa, India) was applied. In group III (EMLA): 2.5 gm of EMLA cream (2.5% lignocaine and 2.5% of prilocaine in 5 gm tube, NEON Laboratories Ltd., Mumbai, India) was applied. All patients were premedicated with tablet lorazepam 2 mg the night before surgery and again at 6 AM of surgery with sips of water.

Depending upon the results of the randomization, an anesthesiologist not involved in the study applied the cream to a 10 cm2 area (site of venous cannulation) on the dorsum of the non-dominant hand of patients in the pre-operative area 1 hour before shifting them to the operation theater (OT). The site was then covered by an identical occlusive dressing. Patients were asked to report if they felt any abnormal sensation of burning, cold, or stinging at the site of cream application and were followed up at 0, 2, and 6 hours for the same. The study was conducted between 9 AM and 11 AM to minimize the influence of circadian difference on pain sensitivity [20].

Before shifting the patients to the theater, the occlusive dressings were removed and the area for venous cannulation was marked. The diameter of the vein before application as well as one-hour post-application was noted by ultrasonography (USG). This procedure was performed using a linear probe of 13 MHz in a cross-sectional view. All participants were scanned for the diameter of the vein by the same senior anesthesiologist trained in using an ultrasound machine for the above procedure. In the OT, cannulation was performed in all patients following manual venous occlusion by another senior expert anesthesiologist using an 18-G cannula and was fixed in place by a transparent dressing. Patients who could not be cannulated in the first attempt were dropped from the study. The primary outcome was the assessment of the severity of pain following venous cannulation, which was done by asking patients to grade the severity of the venipuncture pain experienced on 0-10 VAS, where 0 means no pain and 10 means worst imaginable pain. The secondary outcome was the success rate of cannulation in the first attempt, which predicted ease of cannulation.

The significance of the difference in age, weight, and height was compared by one-way analysis of variance (ANOVA). Data regarding pain (VAS) was compared with a test of proportions (Z test). The package Statistical Package for the Social Sciences (SPSS) v16.0 (SPSS Inc., Chicago) was used for statistical analysis, and p < 0.05 was considered significant.

## Results

Out of 35 patients selected in each group, 10 patients from group I, eight patients from group II, and two patients from group III were excluded from the study as they could not be cannulated in the first attempt. Therefore, the final analysis was carried out on 25 patients, 27 patients, and 33 patients in group I, group II, and group III, respectively. The baseline characteristics such as age, weight, height, and BMI were comparable in all three groups (Table 1).

**Table 1:** Baseline characteristics Values are in mean ± standard deviation (SD).

	Group I (n = 25)	Group II (n = 27)	Group III (n = 33)	p-value
Age (years)	40.57 ± 3.211	41.00 ± 3.387	40.40 ± 3.089	0.727
Height (cm)	156.49 ± 3.776	156.34 ± 4.116	156.00 ± 4.173	0.874
Weight (kg)	80.09 ± 4.883	80.80 ± 4.981	79.46 ± 4.877	0.522
BMI (kg/m^2^)	32.91 ± 4.265	33.20 ± 3.335	32.65 ± 4.114	0.567

The incidence of pain on venipuncture was 100% (25/25 patients) in group I and 92.5% (25/27 patients) in group II, whereas it was significantly reduced in group III, i.e., 60% (20/33 patients). Similar results were found with severity also. The mean VAS was similar in groups I and II, whereas it was significantly reduced in group III (p < 0.05) (Table 2).

**Table 2:** Comparison of the severity of pain among the groups *p < 0.05. Values are in mean ± standard deviation (SD). VAS: Visual analog scale.

VAS	Mean ± SD	f-value	p-value
				Group I	5.97 ± 0.707	209.267	<0.001*
Group II	5.49 ± 1.292						
Group III	1.51 ± 0.702						

The diameter of the vein on which cream was applied was measured before application and 1 hour after application. There was a significant improvement in groups II and III in comparison to group I (p < 0.05) (Table 3).

**Table 3:** Inter-group and intra-group comparisons of the diameter of the vein before (diameter pre) and after application of cream (diameter post) *p < 0.05. Values are in mean ± standard deviation (SD).

	Group I (n = 25)	Group II (n = 27)	Group III (n = 33)	Group I vs II	Group I vs III	Group II vs III
Diameter pre (in mm)	5.48 ± 0.862	5.34 ± 0.639	5.45 ± 0.770	0.5068	0.499	0.8895
Diameter post (in mm)	5.54 ± 0.538	6.74 ± 0.780	5.63 ± 0.490	<0.0001*	0.5094	<0.0001*
p-value	0.5094	<0.0001*	0.2615			

However, there was no significant improvement in ease of cannulation in group II as compared to group I. Eight out of 35 patients (22.85%) in group II could not be cannulated in the first attempt, whereas only two out of 35 patients (5.7%) could not be cannulated in group III, and the difference is statistically significant (p < 0.05) (Table 4).

**Table 4:** Incidences of failure in different groups *p < 0.05. Values are in absolute number or %.

	Group I		Group II		Group III		Group I vs II	Group I vs III	Group II vs III
	No.	%	No.	%	No.	%			
Failure	10	28.6	8	22.9	2	5.7	0.5882	0.0116*	0.0413*

In group II, two patients complained of burning sensation at 0 and 2 hours; however, it was absent at 6 hours. No such adverse effects were seen in group I and group III. The incidences of erythema and blanching were significant in group III at 2 and 6 hours (p < 0.05) (Table 5).

**Table 5:** Comparison of the side effects among different groups Values are in absolute number or %.

Side effects	Time (in hours)	Group I (n = 25)	Group II (n = 27)	Group III (n = 33)
Burning	0	0	2	0
	2	0	2	0
	6	0	0	0
Cold	0	0	0	0
	2	0	0	0
	6	0	0	0
Stinging	0	0	0	0
	2	0	0	0
	6	0	0	0
Blanching	0	0	0	18 (55%)
	2	0	0	4 (12.2%)
	6	0	0	0
Erythema	0	0	0	0
	2	0	0	9 (27.2%)
	6	0	0	1 (3%)
Swelling	0	0	0	0
	2	0	0	0
	6	0	0	0

## Discussion

Pregnancy and parturition are associated with an increase in maternal pain thresholds. This increase in antinociception is partly mediated by a spinal cord dynorphin/kappa opiate receptor system. During pregnancy, the increased maternal antinociception parallels with an increase in spinal dynorphin levels. This opioid analgesia system can be modulated by ovarian sex steroids. This has been observed following the administration of 17-beta-estradiol (E2) and progesterone (P) to non-pregnant rats. The blood concentration of these hormones in non-pregnant rats was matched to pregnancy blood levels. [21]. This resulted in a significantly elevated threshold for pain that closely resembles that of an actual pregnancy. Based on this antinociceptive property of the estradiol, we planned our study. However, to our surprise, we did not find any positive results, i.e., reduction in VAS score during venipuncture in the patients as compared to the control group (p > 0.05). Patients on whom EMLA cream was applied showed a significant decrease in VAS score which is in line with previous literature [15] and significantly better than both the control group and estradiol group. The negative result of estradiol can be explained by various causes. First, there might be incomplete topical penetration of the drug. Second, the dose that we used may be inadequate than that required to produce antinociception. Third, patient factors such as obesity might have resulted in erratic absorption of the drug into the dermis.

The second observation in our study was the change in diameter of the vessel after application. Estrogen mediates cutaneous vasodilation [17], thus increasing the diameter of the vessel. Similarly, EMLA cream is also known to cause vasodilation and erythema [22], although initially, it may cause transient vasoconstriction and blanching. The increase in diameter of the vein was statistically significant in the estradiol group as compared to group placebo and EMLA group (p < 0.05). Though we found that the diameter of the vessel increased in the estradiol group, surprisingly, it did not increase the ease of cannulation as compared to the EMLA group (p < 0.05). This higher failure rate during the cannula placement in the first attempt may be attributed to the lack of cooperation from the patient because of pain.

Estradiol cream's common side effects on vaginal applications are nausea, vomiting, stomach pain, headache, vaginal itching or discharge, breast tenderness, swelling, bloating, and weight gain. Such side effects were not observed in our patients. This may be because just a single-time skin application may not cause enough absorption into the systemic circulation to exert such side effects. Only two patients in the estradiol group complained of burning sensation at 1 hour and 2 hours after application.

In the EMLA group, 55% of patients complained of blanching at 0 hours post-application of cream, and 18% of the patients complained the same at 2 hours, which is in concordance with the previous study [22].

We observed that although estradiol is known for its antinociceptive property, it did not provide effective analgesia during venipuncture. However, due to differences in cutaneous blood flow, skin thickness, and fixed concentration of drug used, it is premature to conclusively comment anything. Further studies with a varied concentration of the drug may provide additional input. Also, the plasma concentration of the drug should have been taken into account. The limitation of our study is we used a fixed concentration of the drug, and the plasma concentration of the drug was not measured.

## Conclusions

There is no beneficial effect of estradiol cream in reducing the pain from venipuncture or in ease of cannulation in comparison to placebo and EMLA cream. EMLA cream was found to significantly reduce the pain of venipuncture in comparison to placebo and estradiol cream. However, as this is a pilot study and considering the few limitations of our study, it may be inconclusive. Further studies taking due care of the limitations of this study are required for a better conclusion.
